# Arsenic Trioxide Sensitizes Glioblastoma to a Myc Inhibitor

**DOI:** 10.1371/journal.pone.0128288

**Published:** 2015-06-03

**Authors:** Yayoi Yoshimura, Akihiko Shiino, Kazue Muraki, Tadateru Fukami, Shigeki Yamada, Takeshi Satow, Miyuki Fukuda, Masaaki Saiki, Masato Hojo, Susumu Miyamoto, Nobuyuki Onishi, Hideyuki Saya, Toshiro Inubushi, Kazuhiko Nozaki, Kenji Tanigaki

**Affiliations:** 1 Research Institute, Shiga Medical Center, Moriyama 5-4-30, Shiga 524–8524, Japan; 2 Department of Neurosurgery, Shiga Medical Center, Shiga 524–8524, Japan; 3 Biomedical MR Science Center, Shiga University of Medical Science, Shiga 520–2192, Japan; 4 Department of Neurosurgery, Shiga University of Medical Science, Shiga 520–2192, Japan; 5 Department of Neurosurgery, Graduate School of Medicine, Kyoto University, Kyoto 606–8507, Japan; 6 Division of Gene Regulation, School of Medicine, Keio University, Tokyo 160–8582, Japan; The Ohio State University, UNITED STATES

## Abstract

Glioblastoma multiforme (GBM) is associated with high mortality due to infiltrative growth and recurrence. Median survival of the patients is less than 15 months, increasing requirements for new therapies. We found that both arsenic trioxide and 10058F4, an inhibitor of Myc, induced differentiation of cancer stem-like cells (CSC) of GBM and that arsenic trioxide drastically enhanced the anti-proliferative effect of 10058F4 but not apoptotic effects. EGFR-driven genetically engineered GBM mouse model showed that this cooperative effect is higher in EGFRvIII-expressing *INK4a/Arf^-/-^* neural stem cells (NSCs) than in control wild type NSCs. In addition, treatment of GBM CSC xenografts with arsenic trioxide and 10058F4 resulted in significant decrease in tumor growth and increased differentiation with concomitant decrease of proneural and mesenchymal GBM CSCs *in vivo*. Our study was the first to evaluate arsenic trioxide and 10058F4 interaction in GBM CSC differentiation and to assess new opportunities for arsenic trioxide and 10058F4 combination as a promising approach for future differentiation therapy of GBM.

## Introduction

Glioblastoma multiforme (GBM) is one of the most common malignant primary brain tumors in adult and highly resistant to conventional chemotherapy[1,2]. Constitutive-active mutation of EGFR (EGFRvIII) and loss of *CDKN2a (INK4a/Arf)* are often observed in GBM[3–7]. Constitutive EGFR activation is sufficient to transform *INK4a/Arf*-deficient neural stem cells (NSCs) and astrocytes into cancer stem-like cells (CSCs), which has high tumorigenicity *in vivo*[8]. GBM CSCs are considered to be an origin of tumor and recurrence [9] and are an important therapeutic target. CSCs can be cultured in serum-free medium supplemented with bFGF and EGF in sphere conditions [10]. When transplanted to immunodeficient mice, CSCs gave rise to high-grade gliomas with pathological phenotypes more similar to GBM compared with GBM cells cultured in serum-containing media [10].

Arsenic trioxide (As_2_O_3_) is used for treatment of acute promyelocytic leukemia (APL), which causes the induction of differentiation of leukemic cells [11,12]. Arsenic trioxide has been reported to inhibit but not regress the growth of a wide variety of solid tumors including GBM at a clinically safety dose (1–2μM). Higher concentrations (10–50μM) are required to induce apoptosis [13–18]. 4μM arsenic trioxide reduces the number of CSCs of GBM, whereas the low concentrations of arsenic trioxide only inhibits renewal of CSCs without affecting properties as CSCs[18]. Thus, new method to enhance its efficacy requires to be exploited. Another candidate target of GBM therapy is c-Myc, which is also required for the maintenance of CSC of various cancers [19]. We show here that a low concentration (2μM) of arsenic trioxide could induce differentiation of GBM CSCs and enhanced the effect of 10058F4, a Myc inhibitor. This interation was grater in EGFRvIII-expressing *INK4a/Arf*-deficient neural stem cells (NSCs) than in wild type NSCs, which suggests a possibility of the application of this combination effect to a therapy of GBM.

## Materials and Methods

### Primary tumor cultures

Primary GBM surgical specimens were obtained from patients undergoing surgical treatment for newly diagnosed GBM at Shiga Medical Center. This study was approved by the Ethical Committee of the Human Research of Shiga Medical Center. The details of the study were explained to patients before they underwent surgery at Shiga Medical Center. Written agreement had been obtained from a patient for the use of the resected tissue for this research, and de-identified tissues were subjected to the analyses in this study. Within 1 h after surgical removal, tumors were washed and enzymatically dissociated into single cells using a neural cell dissociation kit containing papain (Miltenyi Biotec). CSC sphere cells were cultured in NeuroCult NSC Basal Medium (StemCell Technologies, Vancouver, BC, Canada) containing NeuroCult NSC proliferation Supplements (StemCell Technologies), recombinant bFGF and EGF (40 ng/ml each; Peprotech). RI01 and RI02 lines were derived from a 67-year-old man and a 72-year-old man with glioblastoma multiforme, respectively. RI03 and RI08 lines originated from a 77-year old woman, and 57-year-old woman with glioblastoma multiforme, respectively.

Primary CSC neurospheres were dissociated every 4 to 7 days to facilitate cell growth. To promote differentiation, CSC neurospheres were enzymatically dissociated, seeded on a Matrigel-coated slide chamber (Nunc) cultured in the same medium without bFGF and EGF but in the presence of 1% FCS for 1 or 3 days. All primary cells were used at low (<30) passage number.

### Neural stem cell cultures

Normal neural stem cells were isolated from the ganglionic eminences of E14.5 *INK4/Arf* ^*-/-*^ and control embryos. Cells were cultured in NeuroCult NSC Basal Medium (StemCell Technologies, Vancouver, BC, Canada) containing Neurocult NSC proliferation Supplements (StemCell Technologies), recombinant bFGF and EGF (40 ng/ml each; Peprotech). These cells were subsequently infected with EGFRvIII expressing MSCV retrovirus at first passage and treated with puromycin (0.25μg/ml) 48 h after infection. To promote differentiation, EGFRvIII-expressing *INK4/Arf* ^*-/-*^ neural stem cells were dissociated from neurospheres, seeded on a Matrigel-coated slide chamber (Nunc) and cultured in the same medium without bFGF and EGF but in the presence of 1% FCS. Cells were then fixed with 4% PFA for 15 minutes and processed for immunohistochemistry.

### Reagents

10058-F4 (Sigma-Aldrich, St Louis, MO) was dissolved in dimethyl sulfoxide. Arsenic trioxide (Sigma Chemical Co. St. Louis, MO) was dissolved in 5 M solution of sodium hydroxide and then its pH was adjusted to 8.0 with hydrochloric acid. The prepared concentrated solutions were added to the culture medium and mixed gently.

### Cell viability and Caspase 3/7 assay

Cell viability and caspase3/7 activity were determined using PrestoBlue Cell Viability Reagent and CellEvent Caspase-3/7 Green Detection Reagent (Molecular Probes, Invitrogen), respectively. 12 h before drug treatment, cells were seeded at a density of 1x10^4^ cells (100μl) per well in a 96-well plate. The plates were incubated with or without drugs for 24, 72, 168 hours. 10μl of PrestoBlue and 0.2μl of Caspase-3/7 Green Detection Reagent were added to each well and incubated for 30 minutes at 37°C. Fluorescence intensity was determined using a Varioscan Flash plate reader (Thermo Fisher) with an excitation wavelength of 540 nm and an emission wavelength of 590 nm, and an excitation wavelength of 502 nm and an emission wavelength of 530 nm,.

### Immunohistochemistry of tissue sections

Immunohistochemical staining was performed with primary antibodies for 12h at 4°C after blocking for 1 h at room temperature with 5% donkey serum (Millipore). Then the sections were incubated for 1 h at room temperature with secondary antibodies (Molecular Probes). Primary and secondary antibodies used were anti-Nestin (Abcam), anti-Olig2 (IBL), anti-CD44 (SantaCruz), anti-Tuj1 (R&D), anti-Ki67 (Abcam), anti-GFAP (DAKO) and Alexa488 or Alexa594-conjugated donkey anti mouse or rabbit IgG (Invitrogen). The TUNEL assay was performed using the Apoptag Fluorescein *In Situ* Apoptosis Detection Kit (Millipore). Slides were analyzed with a Leica confocal laser scanning microscope (SP8, Leica).

### Animal xenografts and tumor volume measurement

For in vivo experiments, CSCs (5 × 10^4^ cells) were implanted intracranially into 10 week-old female C.B17-lcr SCID mice (Charles River). Two months after transplantation, tumor growth was monitored by animal magnetic resonance imaging (MRI) (7.0 T horizontal-bore MR scanner (Unity Inova; Agilent Technologies, Santa Clara, CA). T2-weighted magnetic resonance imaging was performed in TR/TE 1800 /42 ms with 0.8 mm interval. The sizes of brain tumors were measured in the images. Tumor areas were circumscribed on T2-weighted images using ImageJ (http://imagej.nih.gov/ij/) and the total tumor volume is the sum of their corresponding areas in cm^2^ multiplied by the MR interplane gap of 0.8 mm. Four days after tumor size measurement, Arsenic Trioxide (2.5 mg/kg), 10058F4 (25mg/Kg) or both were administered to the animals by i.p. injection once a day for 10days. After 10-day drug treatments, tumor sizes were again measured using animal MRI, and they were perfused with 4% PFA, and their brains were removed and processed for analysis. For the purpose of histological tumor volume estimation, the brains were cut into 30μm sections and stained with hematoxylin and eosin (HE). Sections were selected at an interval of 210μm. Tumor areas were measured using ImageJ. Tumor volumes were calculated by summing the tumor areas of these sections multiplied by the cross-sectional interval (210μm). The institutional animal care and use committee of Shiga Medical Center approved all of the experiments in our study (Permit number: 24–3, 25–3).

### Gli reporter gene assay

GBM CSCs were transfected with a Gli luciferase reporter construct (Cignal Reporter Assay kits) (SA Biosciences, Frederick, MD, USA) using Lipofectamine2000 (Invitrogen). The medium was replaced with NeuroCult NSC Basal Medium with or without 2μM arsenic trioxide or 60μM 10058F4. After 24 hrs, cells were subjected to luciferase assay using a luminometer (Varioskan Flash). Normalized luciferase activity (firefly luciferase / sea urchin luciferase ratio) was then compared in each experiment, samples were analyzed in triplicate, and experiments were repeated at least three times.

## Results

### Arsenic trioxide and 10058F4 induced differentiation of patient-derived GBM CSCs

**Fig 1:**
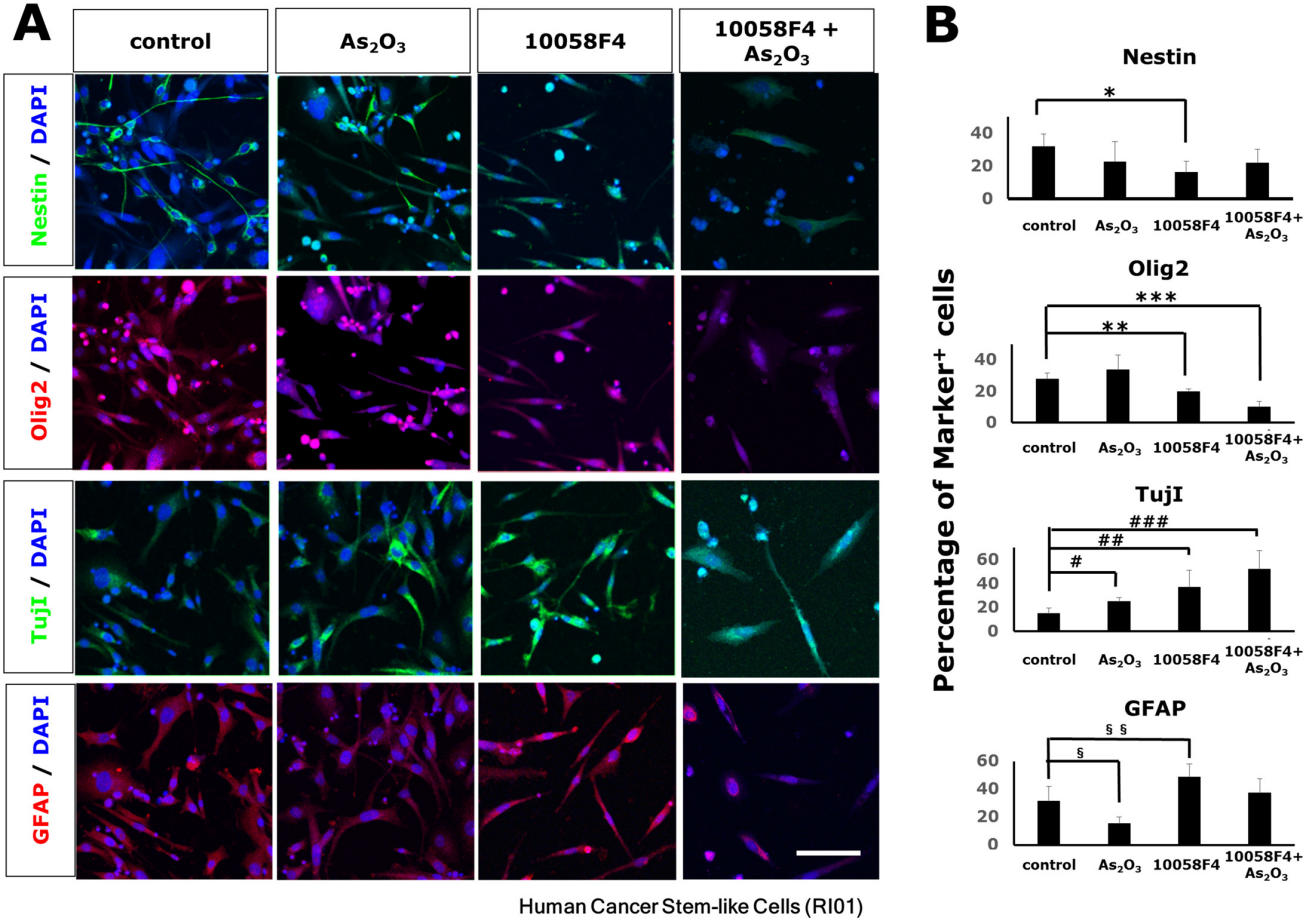
Enhanced differentiation of cancer stem-like cells (CSCs) of glioblastoma multiforme by arsenic trioxide and 10058F4. Immunofluorescent analysis for Nestin (green), Olig2 (red), TujI (green), GFAP(red) and DAPI staining of nuclei (blue) (A) and quantitative analysis of Nestin-positive, Olig2-positive, Tuj1-positive, GFAP-positive cells (B) in cancer stem-like cells (CSCs) of glioblastoma multiforme (GBM) (RI01) 3days after treatment with or without 2μM arsenic trioxide or 60μM 10058F4 in the presence of 1% FCS. Scale bar = 100μm. The data is the mean ± S.D. *P = 0.021, **P = 0.012, ***P = 0.00055, ^##^P = 0.024, ^###^P = 0.0036, ^§^P = 0.031, ^§§^P = 0.050.

To confirm previous reports and examine the effects of arsenic trioxide and 10058F4, an inhibitor of c-Myc on the differentiation of a newly derived GBM CSC neurosphere line (RI01; Materials and Methods), we treated dissociated neurospheres with 2μM arsenic trioxide or 60μM 10058F4 for three days in differentiating condition. Immuno-fluorescent studies showed that 10058F4 reduced the number of Nestin-positive and Olig2-positive cells and increased GFAP-positive cells (Nestin: P = 0.021, Olig2: P = 0.012, GFAP: P = 0.05 (Fig 1A and 1B). In contrast, arsenic trioxide decreased the number of GFAP-positive cells (P = 0.031) (Fig 1A and 1B). Astonishingly, 2μM arsenic trioxide as well as 10058F4 enhanced differentiation of CSCs to TujI-positive cells (arsenic trioxide: P = 0.011, 10058F4: P = 0.024, 10058F4-arsenic trioxide: P = 0.0036) (Fig 1A and 1B). These observations were also confirmed by western blotting (S1A Fig). Similar results were also noted in another 3 human GBM CSC lines (RI02, RI03 and RI08).

### Arsenic trioxide enhanced inhibitory effects of 10058F4 on GBM CSC growth

**Fig 2:**
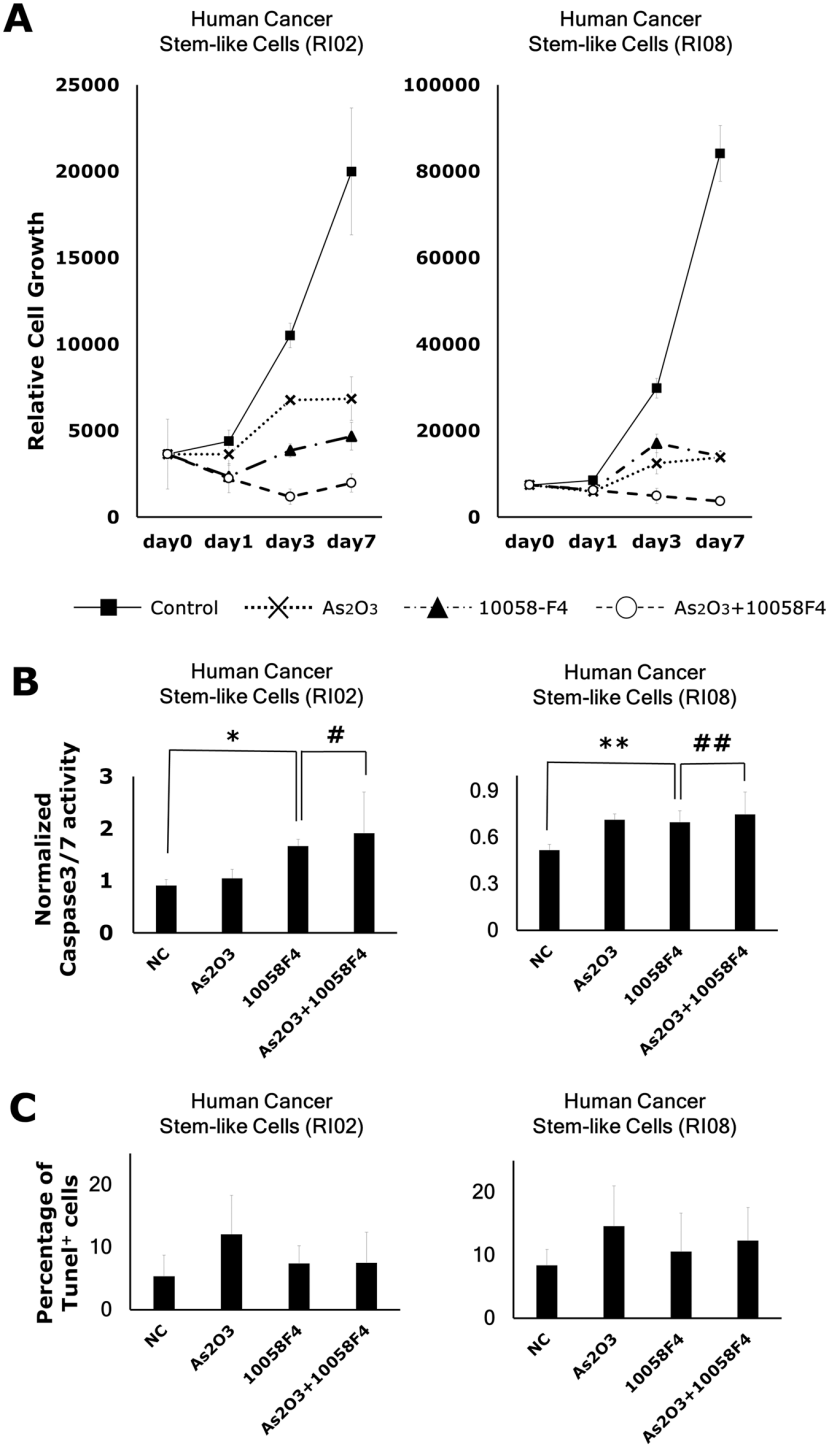
Arsenic trioxide enhanced the inhibitory effect of 10058F4 on GBM CSC growth but not the apoptosis-inducing effect of 10058F4. GBM CSCs (RI02, RI08) were treated with or without 2μM arsenic trioxide or 60μM 10058F4 for 24, 72 and 168h in the presence of 1% FCS. Results are presented as the relative cell growth (A), the activation of Caspase3/7 (B) and the percentages of Tunel^+^ cells (C) as determined with PrestoBlue Cell Viability Reagent, CellEvent Caspase-3/7 Green Detection Reagent and Tunel assay, respectively. Cell viability is presented as the mean ± SD. The relative fluorescent units of treated cells were normalized to the cell viability and presented as the mean ± SD. *P = 0.017, **P = 0.019, ^#^P = 0.72, ^##^P = 0.43.

To determine whether arsenic trioxide potentiate 10058F4 effects, we treated GBM CSCs with 2μM arsenic trioxide in combination with 60μM 10058F4 in differentiating condition. Cell viability assay showed that co-treatment more effectively inhibited GBM CSC cell growth than monotherapy (RI02: F_2,33_ = 10.81, P = 0.0002, RI08: F_2,33_ = 8.02, P = .0014) (Fig 2A). In contrast, this cotreatment did not affect either activation of Caspase-3/7 in apoptotic signaling (RI02: P = 0.63, RI08: P = 0.63) (Fig 2B) or the percentages of Tunel^+^ cells (RI02: P = 0.97, RI08: P = 0.64) (Fig 2C), suggesting this interactive effect is not caused by enhanced apoptosis.

**Fig 3:**
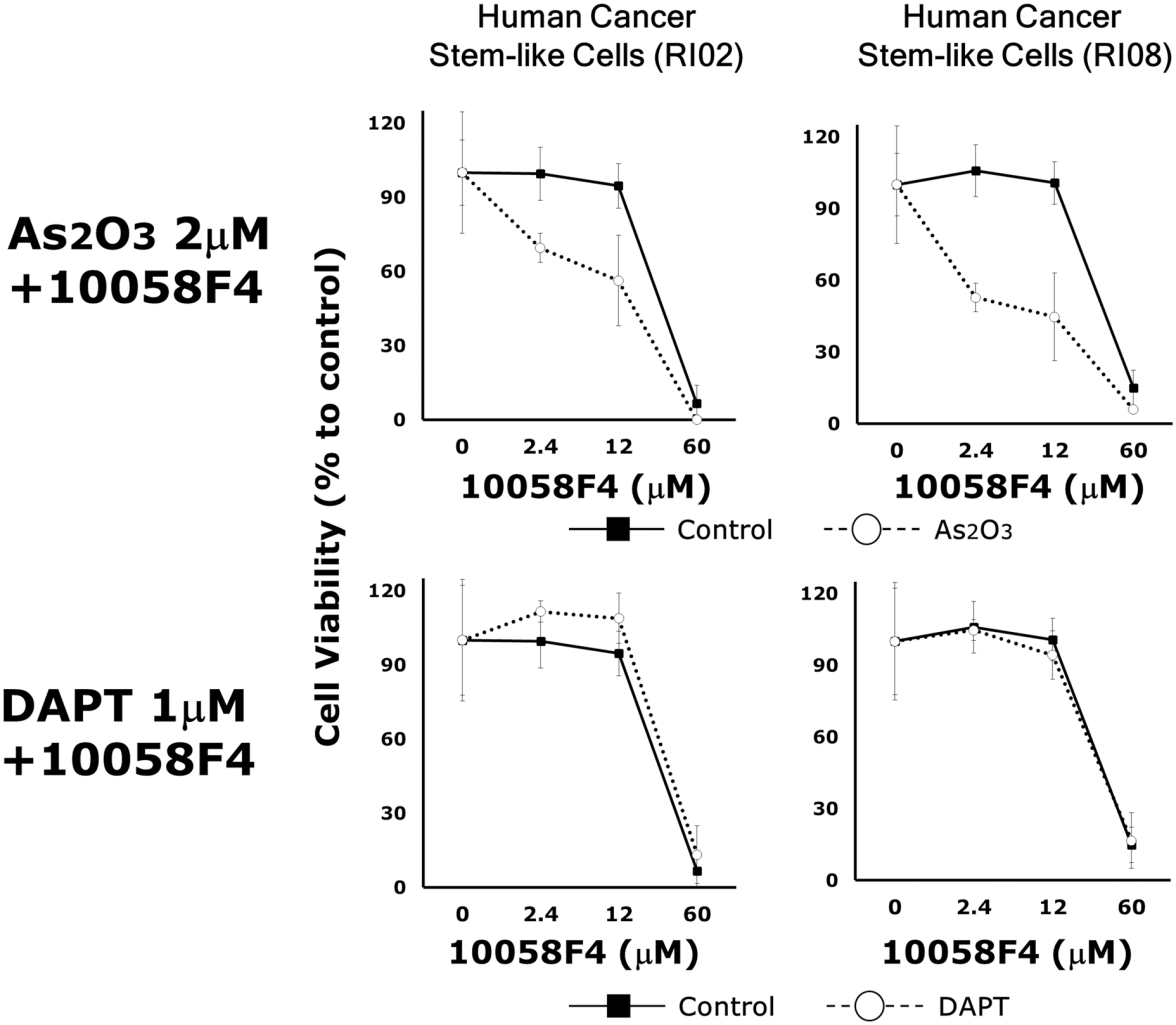
Arsenic trioxide but not DAPT enhanced the responsiveness of GBM CSCs to 10058F4. Dose effects of 10058F4 to GBM CSCs (RI02, RI08) in combination with or without 2μM arsenic trioxide or 1μM DAPT for 72h. Results are presented as the relative cell growth as determined with PrestoBlue Cell Viability Reagent. Cell viability is presented as the mean ± SD.

It has been reported that a constitutive active mutant of Notch could overcome the effect of arsenic trioxide on GBM CSCs [18]. To examine whether Notch signaling is involved in this interactive effects, we subjected GBM CSC lines to a 5x 3 doses of 10058F4 (2.4μM, 12μM, 60μM) singly and in combination with 2μM arsenic trioxide or 1μM DAPT, a Notch signaling inhibitor. The individual and combined survival curve showed that arsenic trioxide but not DAPT enhanced the responsiveness to 10058F4 (RI02: F_1,4_ = 23.20, P = 0.0085, RI08: F_1,4_ = 1950.419, P = 0.00001) (Fig 3). Similar results were also obtained in another 2 GBM CSC lines (RI01, RI03).

**Fig 4:**
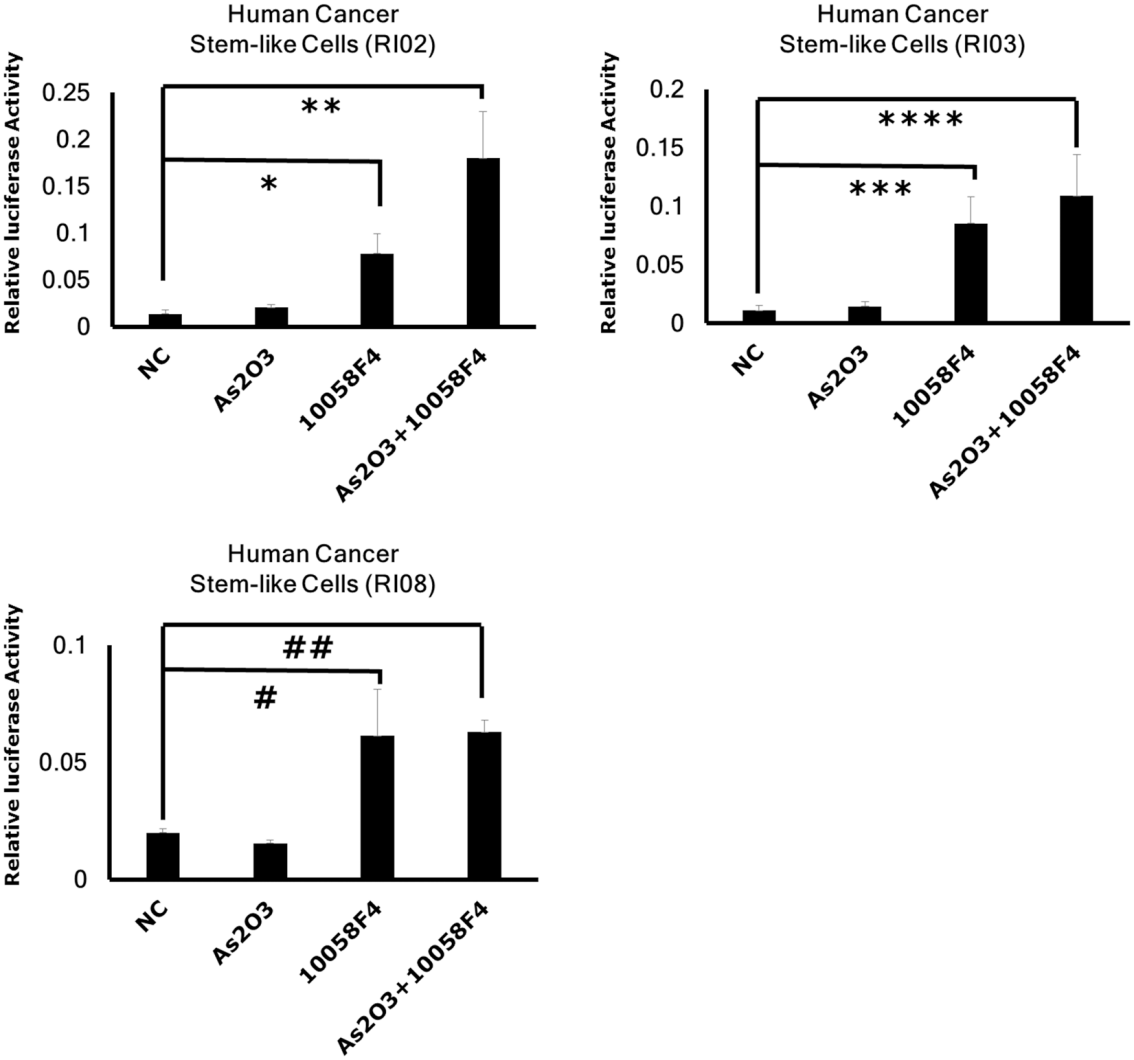
10058F4 but not arsenic trioxide activated Shh signaling in GBM CSCs. GBM CSCs were transfected with a reporter construct containing a response element for Gli. After 24 hour 10058F4 (60μM) and arsenic trioxide (2μM) treatment, luciferase assay was performed. Results are the mean ± S.D. of triplicate data points from a representative experiment. *P = 0.0065 ** P = 0.0043, ***P = 0.0050, ****P = 0.0091, ^#^P = 0.023028, ^##^P = 0.00015.

Shh signaling is also essential for the maintenance of GBM CSCs and GBM cells often become resistant to Notch inhibitor through the activation of Shh signaling [20,21]. Next, we examined the effects of arsenic trioxide and 10058F4 on Shh signaling in GBM CSCs. Gli reporter assay showed that 10058F4 activated Shh signaling in GBM CSCs but arsenic trioxide did not enhance its effect (Fig 4). Taken together, the effect of this combination therapy seems to be independent of Notch/ Shh signaling. However, off-target effects of these inhibitors are not excluded in this study.

### Loss of *Ink4a/Arf* and constitutively activated EGFR enhanced the responsiveness to arsenic trioxide and 10058F4

**Fig 5:**
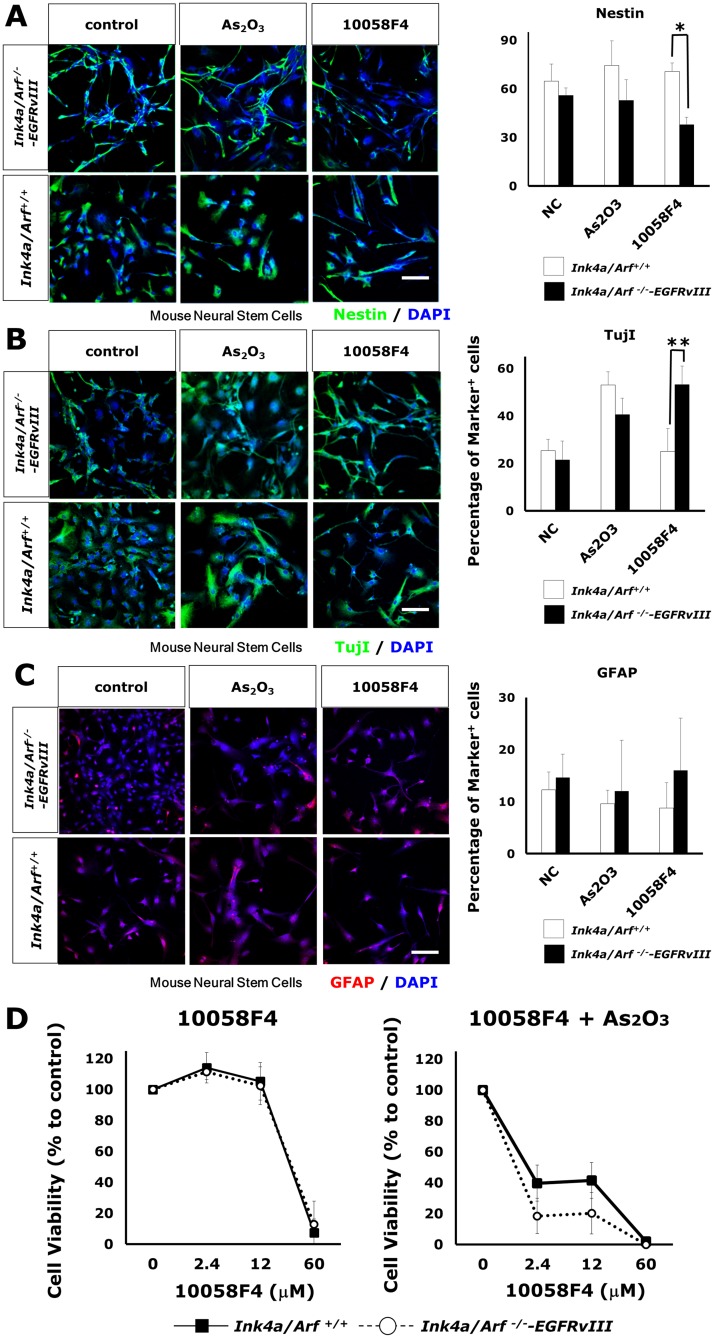
Constitutive activation of EGFR with loss of *Ink4/Arf* enhanced the responsiveness of neural stem cells to arsenic trioxide and 10058F4. (A)-(C) Immunofluorescent analysis for Nestin (green, (A)), TujI (green (B)), GFAP (red (C)) and DAPI staining of nuclei (blue) and quantitative analysis of Nestin-positive(A), Tuj1-positive cells (B), GFAP-positive cells (C) in *Ink4/Arf* ^*-/-*^—EGFRvIII and control neural stem cells 3days after treatment with or without 2μM arsenic trioxide or 60μM 10058F4. Scale bar = 100μm. The data is the mean ± S.D. *P = 0.00001, **P = 0.0041. (D) Dose effects of 10058F4 to *Ink4/Arf* ^*-/-*^—EGFRvIII and control neural stem cells in combination with or without 2μM arsenic trioxide for 72h. Results are presented as the relative cell growth as determined with PrestoBlue Cell Viability Reagent. Cell viability is presented as the mean ± SD.

EGFR activation in *Ink4/Arf*-deficient astrocytes and neural stem cells (NSCs) is known to provoke GBM-like phenotypes[8]. A constitutively active mutant form of EGFR (EGFRvIII) transforms NSCs into tumorigenic and GBM CSC-like cells. This genetically-defined system provides an opportunity to investigate how EGFR activation with a loss of *Ink4/Arf* influences the responsiveness of NSCs arsenic trioxide and 10058F4 in differentiating condition. The differentiation of *Ink4/Arf* ^*+/+*^ NSCs was not affected by 10058F4 (Fig 5A–5C). In contrast, 10058F4 caused an enhanced differentiation of retrovirally EGFRvIII-transduced *Ink4/Arf* ^*-/-*^ NSCs to TujI-positive cells and reduced number of Nestin-positive cells (Nestin: P = 0.00001, TujI: P = 0.0041) (Fig 5A), although arsenic trioxide enhanced TujI-positive cell differentiation of both *Ink4/Arf* ^*+/+*^ NSCs and EGFRvIII-transduced *Ink4/Arf* ^*-/-*^ NSCs (*Ink4/Arf* ^*+/+*^ NSCs: P = 0.00031, EGFRvIII-transduced *Ink4/Arf* ^*-/-*^ NSCs: P = 0.011) (Fig 5B). These results are also confirmed by western blotting (S1B Fig). Cell viability assay showed that EGFR activation and *Ink4/Arf* deficiency increased the sensitivity to co-treatment with arsenic trioxide and 10058F4 but not mono-treatment with 10058F4 (10058F4: genotype x dose interaction, F_3,15_ = 0.53, P = 0.67, arsenic trioxide and 10058F4: genotype x dose interaction, F_3,12_ = 3.95, P = 0.036) (Fig 5D). These findings raise the possibility that arsenic trioxide and 10058F4 combination therapy might be more effective on EGFR-active *Ink4/Arf*-deficient GBM CSCs compared with on normal NSCs.

### Arsenic trioxide and 10058 combination treatment blocked glioma progression in a GBM CSC xenograft model

**Fig 6:**
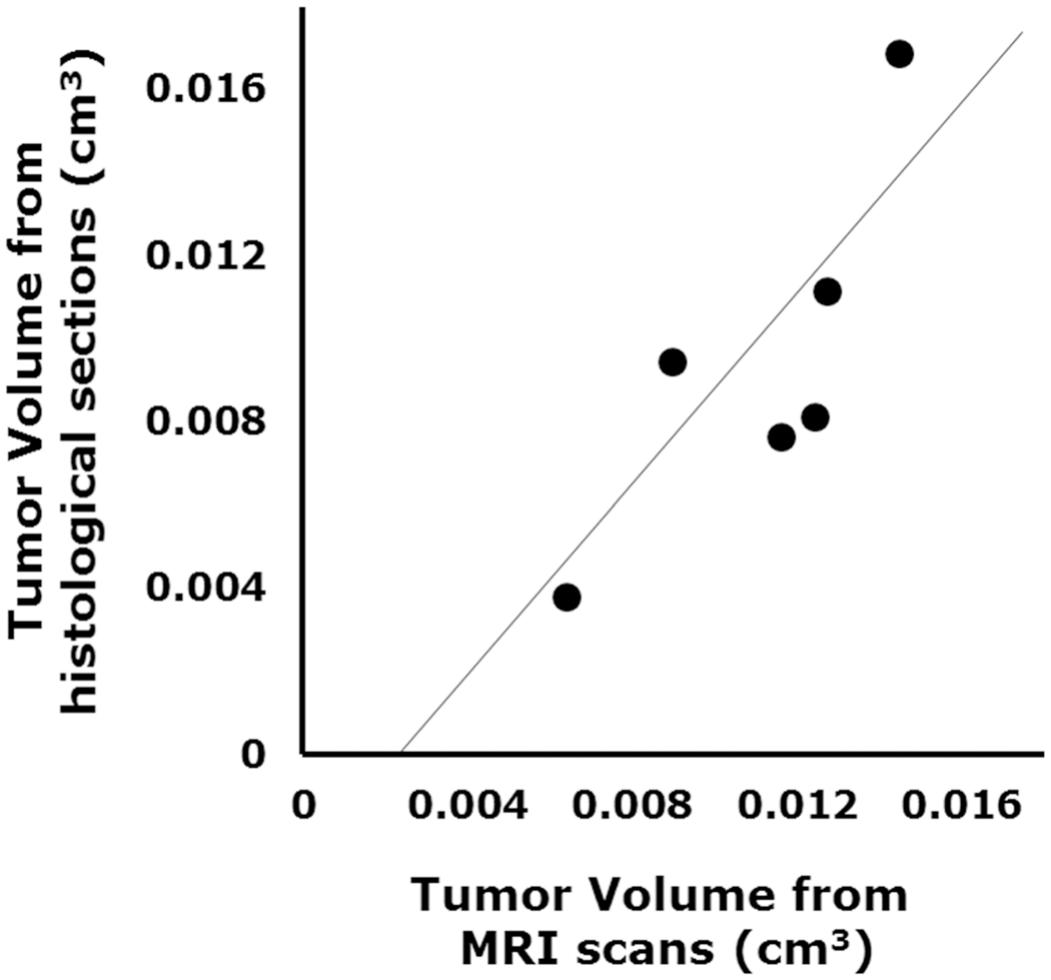
The correlation between tumor volume measurements from MRI scan data and those made from histological sections. GBM CSCs (RI03) CSCs (5 × 10^4^ cells) were implanted intracranially into SCID mice. Two months after transplantation, tumor growth was estimated by MRI and histological sections. The correlation efficient is R = 0.81, P = 0.049.

**Fig 7:**
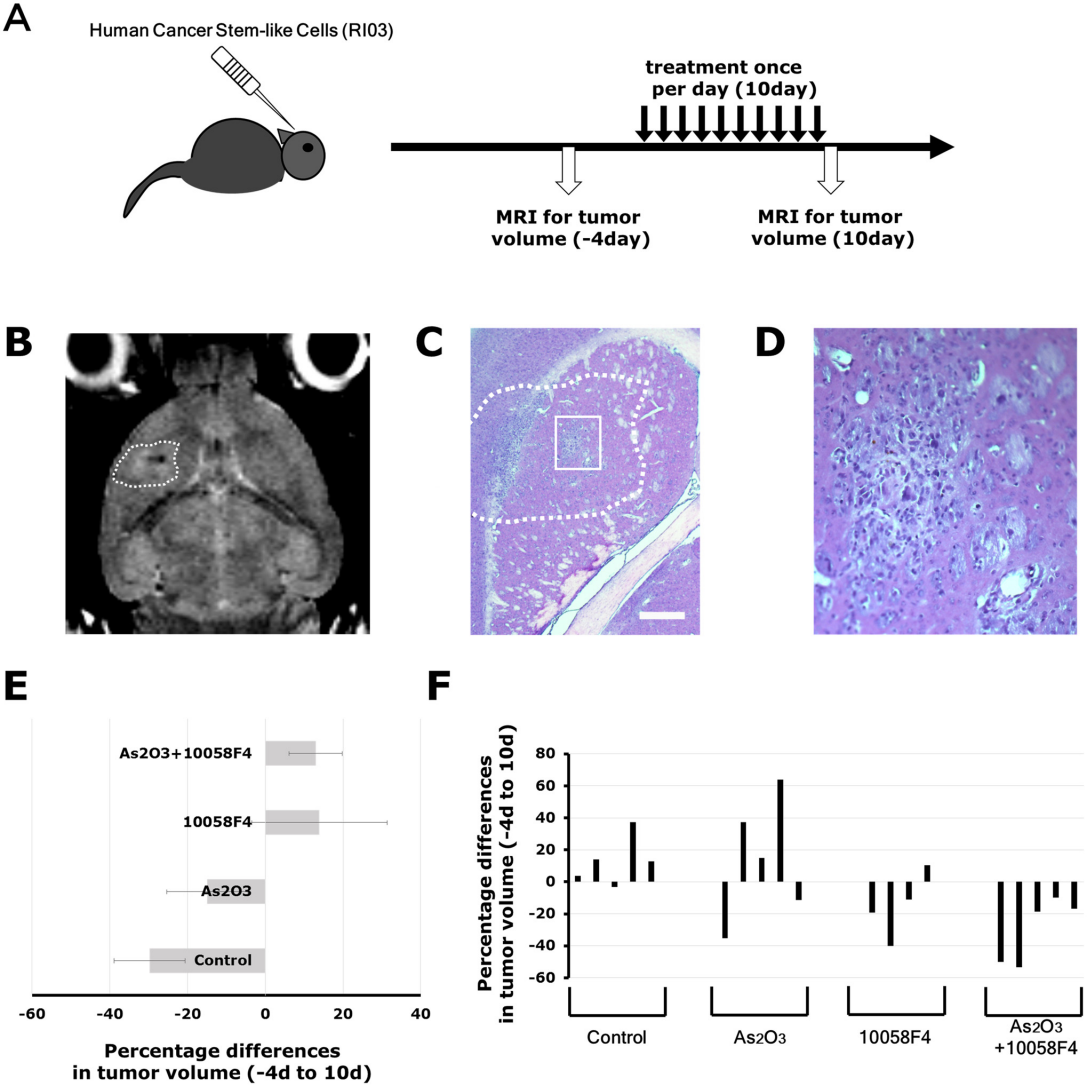
Arsenic trioxide and 10058F4 combination treatment efficiently regressed established gliomas. Experimental Design. GBM CSCs (RI03) CSCs (5 × 10^4^ cells) were implanted intracranially into SCID mice. Two months after transplantation, tumor growth was monitored by MRI. Four days after tumor size measurement, Arsenic Trioxide (2.5 mg/kg), 10058F4 (25mg/Kg) or both were administered by i.p. injection once a day for 10 days. After 10-day drug treatments, tumor sizes were again measured. Representative images of T2-weighted MRI. The region of interest used to calculate the volume of brain tumor is indicated by a dashed line. (C)-(D) Representative photographs of hematoxylin / eosin staining of intracranial xenograft brain tumors. The boxed area in (C) is magnified in (D). Scale bar = 500μm. (E)-(F) Changes in tumor volume after 10-day treatment with arsenic trioxide and 10058F4 relative to the starting tumor volume for each individual mouse. Each bar represents a volume change of an individual mouse. The data in (E) is shown as the mean ± SD of the data for each individual mouse in (F).

**Fig 8:**
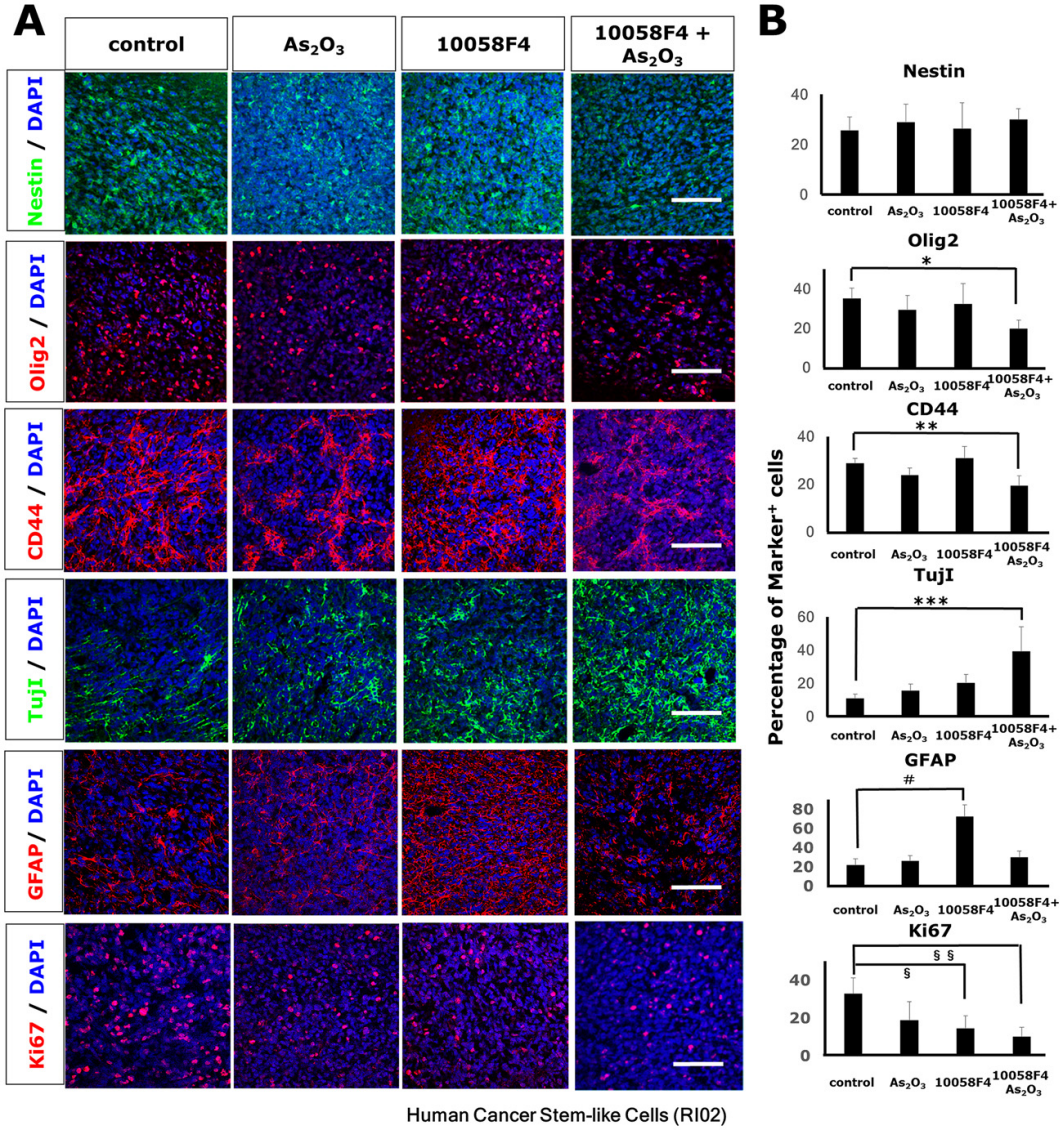
Arsenic trioxide and 10058F4 combination treatment reduced proneural/mesenchymal GBM CSC and induced differentiation to TujI-positive cells in a human GBM CSC xenograft model. SCID mice were injected intracranially with 5 × 10^4^ GBM CSCs (RI02). Treatment with arsenic Trioxide (2.5 mg/kg), 10058F4 (25mg/Kg) or both was initiated after the confirmation of tumor growth with MRI and continued daily for 10days. Representative images from 10-day trial in a human GBM CSC xenograft model (A). Immunofluorescent analysis for Nestin (green), Olig2 (red), CD44 (red), TujI (green), GFAP (re), Ki67 (red) and DAPI staining of nuclei (blue) (A) and quantitative analysis of Nestin-positive, Olig2-positive, CD44-positive, Tuj1-positive, GFAP-positive, Ki67-positive cells (B). Scale bar = 100μm. The data is the mean ± S.D. *P = 0.05, **P = 0.001, ***P = 0.001, ^#^P = 0.00039, ^**§**^P = 0.015847, ^**§§**^P = 0.0042.

To extend our *in vitro* finding, we studied the effect of arsenic trioxide and 10058F4 on *in vivo* tumor growth and progression using a GBM CSC xenograft model. MRI is a promising noninvasive technique to monitor treatment-induced changes in tumor growth in mice *in vivo* [22]. Tumor volumes derived from MR images were well-correlated with tumor volumes estimated by histological sections (R = 0.81, P = 0.049) (Fig 6). We assessed initial tumor growth using MRI two months after intracranial injection of GBM CSCs, and randomized implanted mice into arsenic trioxide, 10058F4, both or vehicle cohorts. After 10-day treatment with arsenic trioxide and 10058F4, we reassessed subsequent tumor growth (Fig 7A). Arsenic trioxide and 10058F4 were well tolerated over 10-day treatment with no visible side effects (S2 Fig), which is consistent with previous reports [23,24]. Arsenic trioxide and 10058F4 combination treatment but not mono-treatment regressed GBM CSC tumor (10058F4: P = 0.13, arsenic trioxide: P = 0.95, 10058F4-arsenic trioxide: P = 0.020, one-way ANOVA, Fisher’s LSD test) (Fig 7B–7F and S3 Fig). To determine the mechanism underlying this marked effects of arsenic trioxide and 10058F4 co-treatment, we performed immunohistochemical analysis of treated tumor samples. 10058F4 and cotreatment with arsenic trioxide decreased Ki67-positive proliferating cells (10058F4: P = 0.016, 10058F4-arsenic trioxide: P = 0.0042) (Fig 8A and 8B). Arsenic trioxide and 10058F4 co-treated mice but not mono-treated mice exhibited drastic reduction of Olig2-positive proneural GBM CSCs and CD44-positive mesenchymal GBM CSCs [25–27] and enhanced differentiation to TujI-positive cells (Olig2: P = 0.027, CD44: P = 0.0075, TujI: P = 0.00093) (Fig 8A and 8B). In addition, 10058F4 increased the number of GFAP-positive cells (P = 0.00039), but arsenic trioxide inhibited the effect (Fig 8A and 8B). Thus these data demonstrated that arsenic trioxide and 10058F4 co-treatment induced differentiation of GBM CSCs *in vivo* with concomitant loss of GBM CSCs.

## Discussion

Clinical developments of arsenic trioxide and 10058F4, a c-Myc inhibitor are limited due to their low effectiveness, although they have important potential. 10058F4 inhibits c-Myc-mediated transactivation *in vitro*, but failed to show its efficiency *in vivo* except for neuroblastoma models[24,28]. Arsenic trioxide is required to be used at higher concentrations (10–50μM) than its safety limit for effective treatment of GBM *in vivo* [14–18]. Our results demonstrated that a low concentration of arsenic trioxide (2μM) enhanced the sensitivity of GBM CSCs to 10058F4 and that arsenic trioxide and 10058F4 combination treatment enhanced differentiation of GBM CSCs. This preclinical efficacy of arsenic trioxide and 10058F4 combination therapy were confirmed across multiple GBM models: human GBM CSCs, genetically-engineered mice GBM model, and human-derived GBM CSC xenografts *in vivo*.

Arsenic trioxide and 10058F4 have been reported to regulate metabolic pathways, which play pivotal roles to balance quiescence and proliferation of various types of CSCs. Maintenance of various stem cells including CSCs relies on anaerobic glycolysis, glutamine metabolism and fatty acid metabolism for their survival and proliferation[29–31]. Stem cells and cancer cells uptake glucose at a high rate and convert the majority of it to lactate[32]. This aerobic glycolysis enables an efficient supply of macromolecules for proliferation. In GBM cells, phosphoinositol 3 kinase (PI3K)/Akt signaling and a constitutive active form of EGFR, EGFRvIII promote this glycolytic metabolism through c-Myc regulation [33–35]. EGFRvIII activates c-Myc signaling through the control of alternative splicing of Max, a Myc binding partner, and mTORC2-dependent upregulation of c-Myc expression level [34,35]. However, when glucose metabolism is limited, glutaminolysis and fatty acid consumption are essential for the survival of cancer cells [36]. Glutaminolysis is also regulated by c-Myc signaling in neuroblastoma cells [36–38]. c-Myc coordinates the expression of the genes necessary for glutaminolysis. c-Myc diverts glucose-derived pyruvate into lactate from tricarboxylic acid cycle (TCA cycle) by induction of lactate dehydrogenase A (LDH-A). As a result, glucose carbon is completely away from mitochondria, and glutamine is essential to maintain TCA cycle activity, which made c-Myc-transformed cells susceptible to inhibition of mitochondrial electron transport chain[36,39], which might explain cooperative effects of 10058F4 with arsenic trioxide, because arsenic trioxide is a mitochondrial toxin[40,41]. A c-Myc regulator, the zinc finger and X-linked transcription factor (ZFX) is indispensable for CSC self-renewal in GBM and AML. ZFX deficiency leads to loss of CSC properties, which can be partially rescued by overexpression of mitochondrial enzymes, Ptpmt1 and Idh2[42,43]. This finding also suggests the important roles of ZFX-induced c-Myc in mitochondrial functions.

Arsenic trioxide has been shown to induce promyelocytic leukemia protein (PML) degradation[12,44]. PML promotes fatty acid oxidation through activation of peroxidase proliferator-activated receptor (PPAR) signaling [45]. Myc can also promote fatty acid synthesis and fatty acid oxidation [24,39,46]. Fatty acid oxidation provides acetyl-coA to maintain TCA cycle. GBM with EGFR activation is highly dependent on fatty acid synthesis and fatty acid oxidation for survival [47,48]. It is tempting to speculate that fatty acid synthesis and oxidation might be another target of arsenic trioxide and 10058F4 combination therapy. PML is a member of the tripartite motif (TRIM) family and involved in protein degradation through SUMOylation and ubiquitination [49]. It has been reported that PML affects stabilization of c-Myc in AML cells [50]. Another member of TRIM, TRIM3 is a tumor-suppressor of GBM [51]. This protein degradation machinery might be another target of arsenic trioxide.

In summary, we demonstrated an effective pharmacological partnership between arsenic trioxide and c-Myc inhibition that enhanced differentiation of GBM CSCs and regressed GBM CSC tumor growth *in vivo*. These observations provide a foundations for further studies of arsenic trioxide-10058F4 combination therapy for GBM.

## Supporting Information

S1 Fig Quantification of Olig2, TujI and β-Actin in GBM CSCs and *Ink4/Arf* ^*-/-*^—EGFRvIII neural stem cells. Western blots showing Olig2, TujI and β-Actin levels in GBM CSCs (RI01) (A) and *Ink4/Arf* ^*-/-*^—EGFRvIII neural stem cells (B)1day after treatment with or without 2μM arsenic trioxide or 60μM 10058F4.
(TIF)

S2 Fig As_2_O_3_ and 10058F4 are well tolerated for 10-day treatment. As_2_O_3_ (2.5 mg/kg) and 10058F4 (25mg/Kg) treatment are well-tolerated for up to 10days (endpoint for this study). Mean weight for 5 male mice over the 10-day trial. Mice were divided by treatment group: control, As_2_O_3_, 10058F4 and both. After the trial, these mice did not show any obvious macroscopic symptoms.
(TIF)

S3 Fig The effects of Arsenic trioxide and 10058F4 combination treatment. (A) Representative images of T2-weighted MRI. The region of interest used to calculate the volume of brain tumor is indicated by a dashed line. GBM CSCs (RI03) CSCs (5 × 10^4^ cells) were implanted intracranially into SCID mice. Two months after transplantation, tumor growth was monitored by MRI. Four days after tumor size measurement, Arsenic Trioxide (2.5 mg/kg), 10058F4 (25mg/Kg) or both were administered by i.p. injection once a day for 10days. After 10-day drug treatments, tumor sizes were again measured. (B)–(C) Representative photographs of hematoxylin / eosin staining of intracranial xenograft brain tumors. The boxed area in (B) is magnified in (C). Scale bar = 500μm.
(TIF)
