# Correction: Arsenic Trioxide Sensitizes Glioblastoma to a Myc Inhibitor

**DOI:** 10.1371/journal.pone.0149826

**Published:** 2016-02-16

**Authors:** Yayoi Yoshimura, Akihiko Shiino, Kazue Muraki, Tadateru Fukami, Shigeki Yamada, Takeshi Satow, Miyuki Fukuda, Masaaki Saiki, Masato Hojo, Susumu Miyamoto, Nobuyuki Onishi, Hideyuki Saya, Toshiro Inubushi, Kazuhiko Nozaki, Kenji Tanigaki

Fig 7 is incorrect. Panel E was incorrectly labeled. The authors have provided a corrected version here.

**Fig 7:**
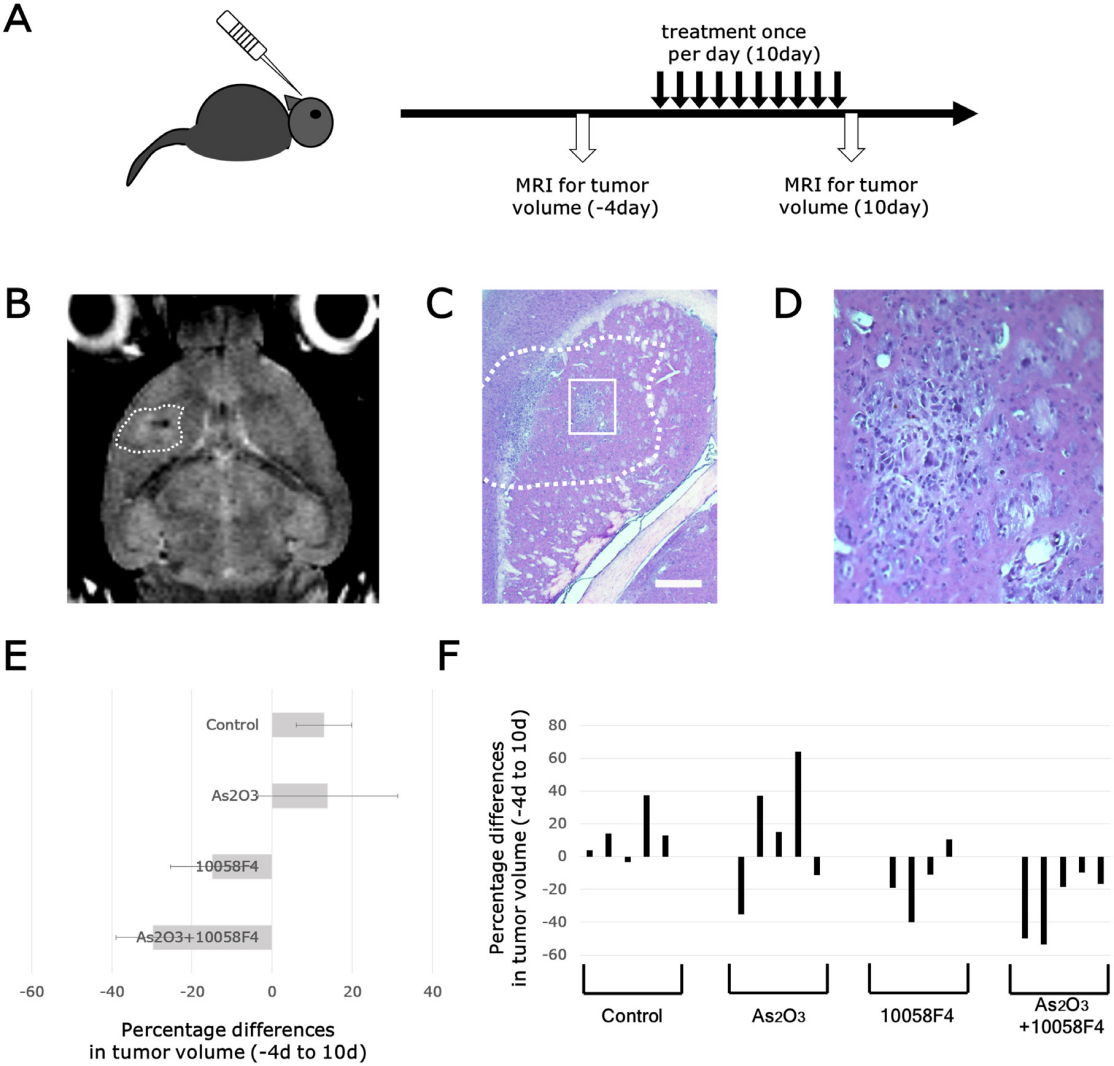
Arsenic trioxide and 10058F4 combination treatment efficiently regressed established gliomas. Experimental Design. GBM CSCs (RI03) CSCs (5 × 104 cells) were implanted intracranially into SCID mice. Two months after transplantation, tumor growth was monitored by MRI. Four days after tumor size measurement, Arsenic Trioxide (2.5 mg/kg), 10058F4 (25mg/Kg) or both were administered by i.p. injection once a day for 10 days. After 10-day drug treatments, tumor sizes were again measured. Representative images of T2-weighted MRI. The region of interest used to calculate the volume of brain tumor is indicated by a dashed line. (C)-(D) Representative photographs of hematoxylin / eosin staining of intracranial xenograft brain tumors. The boxed area in (C) is magnified in (D). Scale bar = 500μm. (E)-(F) Changes in tumor volume after 10-day treatment with arsenic trioxide and 10058F4 relative to the starting tumor volume for each individual mouse. Each bar represents a volume change of an individual mouse. The data in (E) is shown as the mean ± SD of the data for each individual mouse in (F).

## References

[1] Yoshimura Y, Shiino A, Muraki K, Fukami T, Yamada S, Satow T, et al (2015) Arsenic Trioxide Sensitizes Glioblastoma to a Myc Inhibitor. PLoS ONE 10(6): e0128288 doi: 10.1371/journal.pone.0128288 26038891 10.1371/journal.pone.0128288 PMC4454553

