# Supplementary figures and images for: Arsenic Trioxide Sensitizes Glioblastoma to a Myc Inhibitor
Source: PLoS One. 2015 Jun 3;10(6):e0128288. doi: 10.1371/journal.pone.0128288 (PMC4454553; doi:10.1371/journal.pone.0128288)

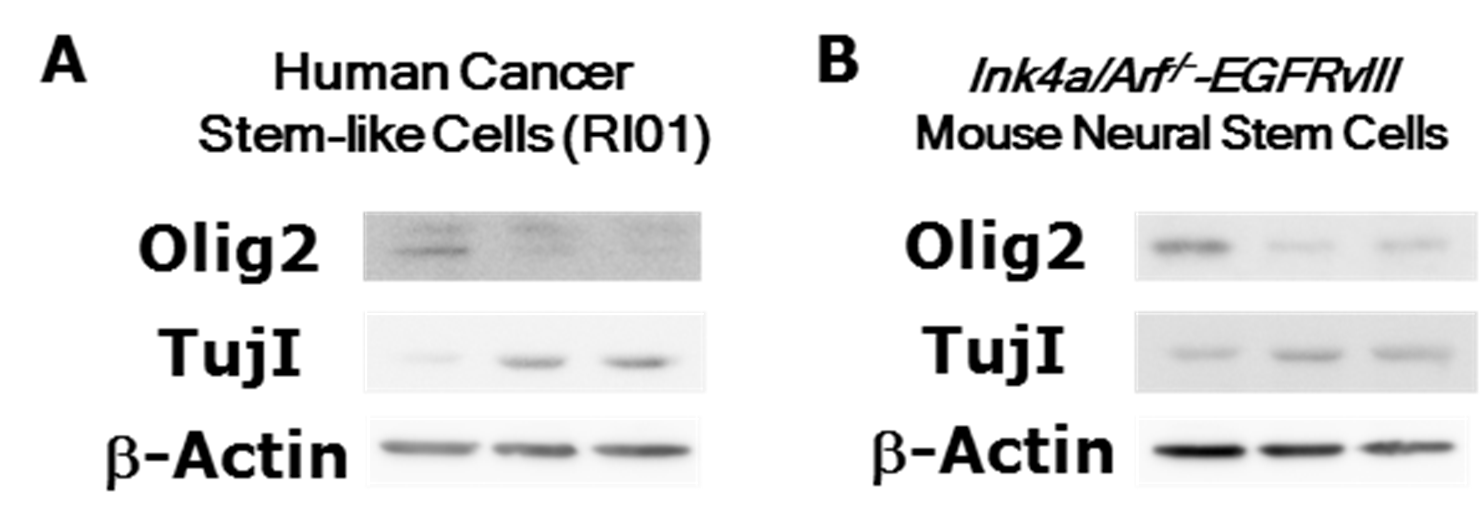

Supplement: S1 Fig — Western blots showing Olig2, TujI and β-Actin levels in GBM CSCs (RI01) (A) and Ink4/Arf -/-—EGFRvIII neural stem cells (B)1day after treatment with or without 2μM arsenic trioxide or 60μM 10058F4. (TIF) [file pone.0128288.s001.tif]

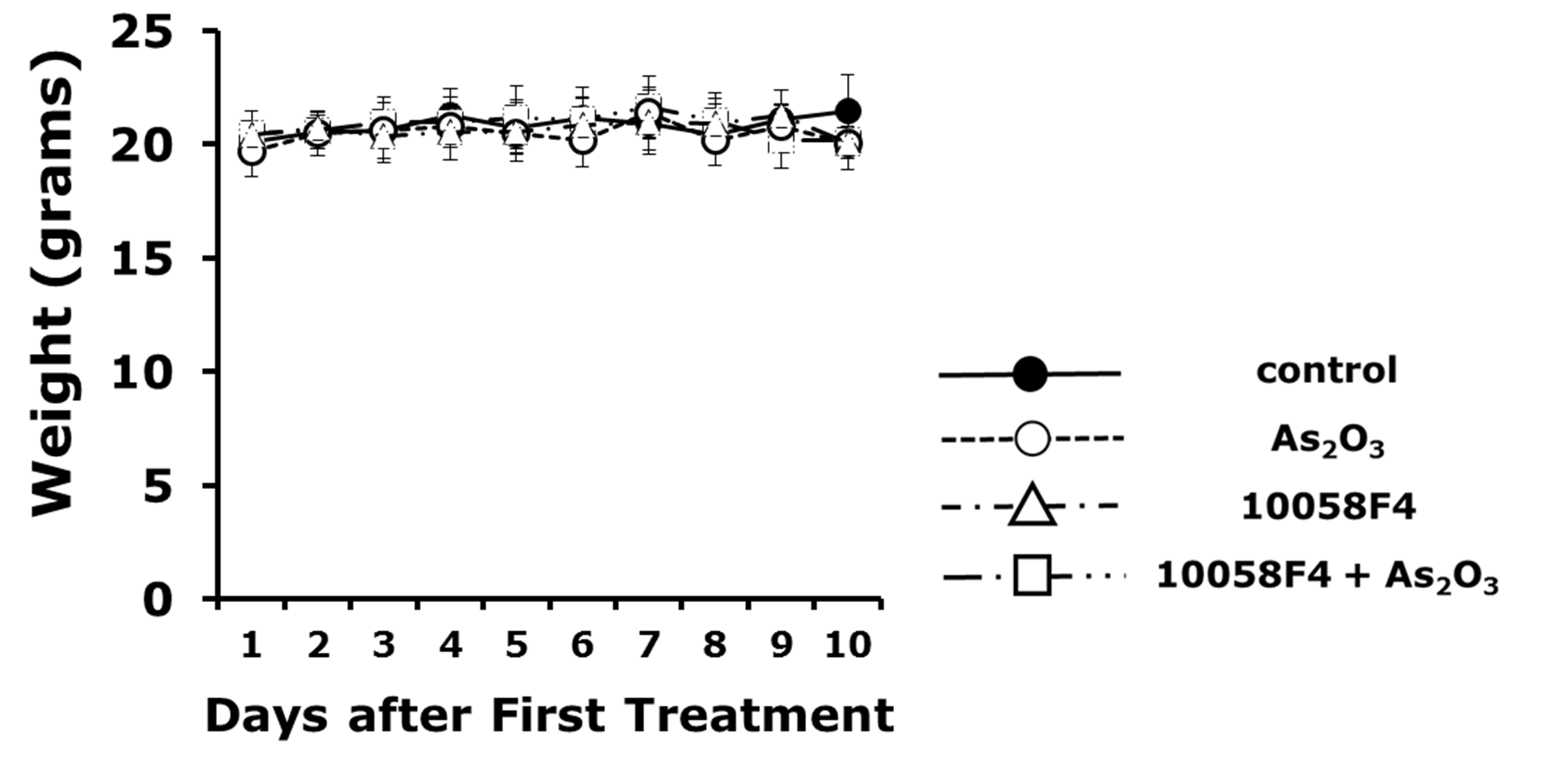

Supplement: S2 Fig — As2O3 (2.5 mg/kg) and 10058F4 (25mg/Kg) treatment are well-tolerated for up to 10days (endpoint for this study). Mean weight for 5 male mice over the 10-day trial. Mice were divided by treatment group: control, As2O3, 10058F4 and both. After the trial, these mice did not show any obvious macroscopic symptoms. (TIF) [file pone.0128288.s002.tif]

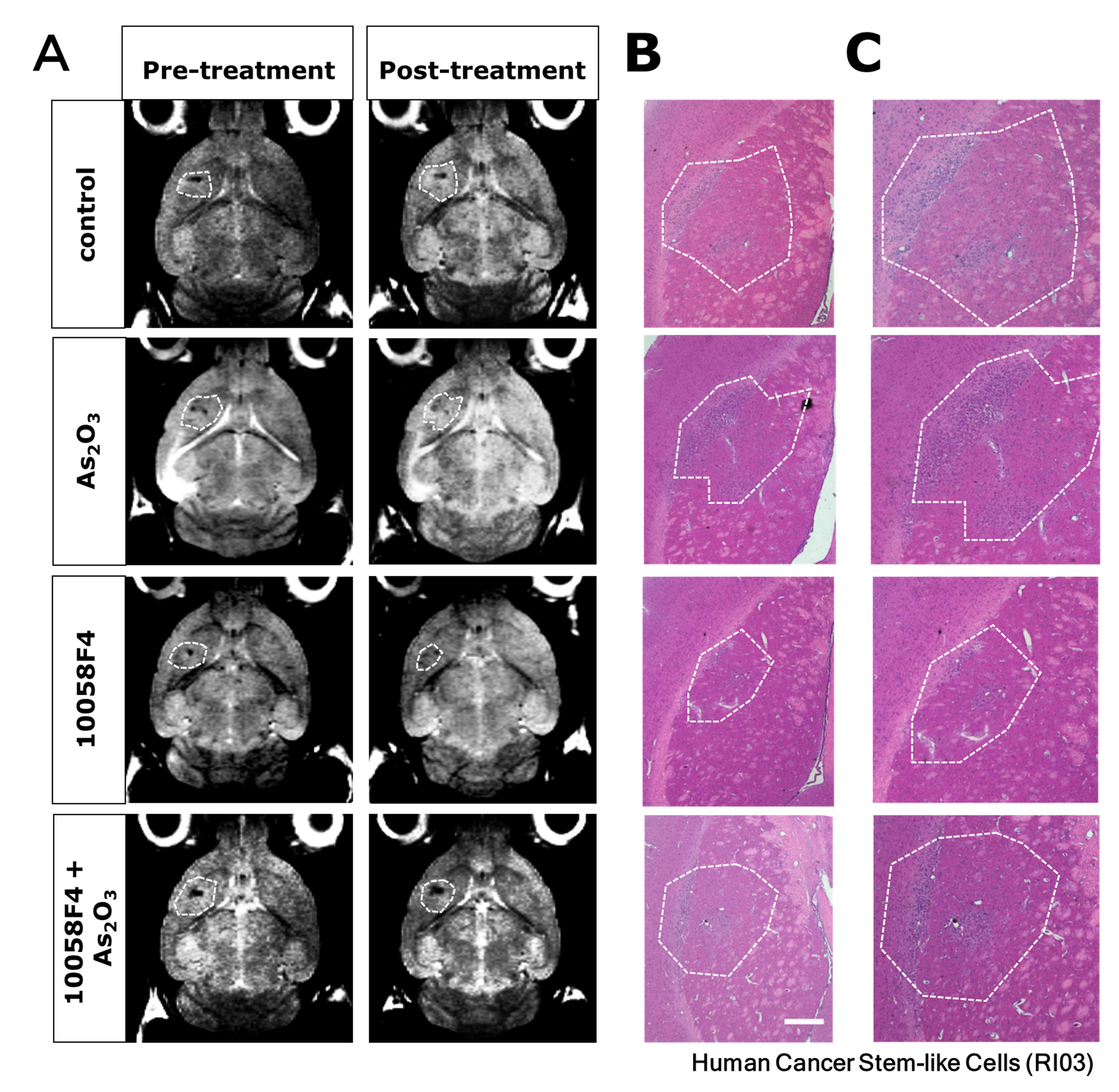

Supplement: S3 Fig — (A) Representative images of T2-weighted MRI. The region of interest used to calculate the volume of brain tumor is indicated by a dashed line. GBM CSCs (RI03) CSCs (5 × 10^4^ cells) were implanted intracranially into SCID mice. Two months after transplantation, tumor growth was monitored by MRI. Four days after tumor size measurement, Arsenic Trioxide (2.5 mg/kg), 10058F4 (25mg/Kg) or both were administered by i.p. injection once a day for 10days. After 10-day drug treatments, tumor sizes were again measured. (B)–(C) Representative photographs of hematoxylin / eosin staining of intracranial xenograft brain tumors. The boxed area in (B) is magnified in (C). Scale bar = 500μm. (TIF) [file pone.0128288.s003.tif]
